# Preimplantation Genetic Testing of Multiple Endocrine Neoplasia Type 2A

**DOI:** 10.3389/fendo.2020.572151

**Published:** 2020-10-14

**Authors:** Anders Würgler Hansen, Laura Kirstine Sønderberg Roos, Kristine Løssl, Christian Godballe, Jes Sloth Mathiesen

**Affiliations:** ^1^Faculty of Health Sciences, University of Southern Denmark, Odense, Denmark; ^2^The Emergency Department, Sydvestjysk Sygehus, Esbjerg, Denmark; ^3^Department of Clinical Genetics, Copenhagen University Hospital Rigshospitalet, Copenhagen, Denmark; ^4^The Fertility Clinic, Copenhagen University Hospital Rigshospitalet, Copenhagen, Denmark; ^5^Department of ORL Head and Neck Surgery and Audiology, Odense University Hospital, Odense, Denmark; ^6^Department of Clinical Research, University of Southern Denmark, Odense, Denmark

**Keywords:** PGT-M, assisted reproductive technology, rearranged during transfection (RET), medullary thyroid carcinoma, multiple endocrine neopasia type 2

## Abstract

**Background:** When discussing matters of reproduction, the 2015 revised guidelines for the management of medullary thyroid carcinoma recommend that patients diagnosed with multiple endocrine neoplasia type 2A (MEN 2A) are informed about the option of Preimplantation Genetic Testing for Monogenic Disorders (PGT-M). In addition, patients seem to have a genuine interest in reproductive options. However, there are just two reports worldwide of this technology being used for patients with MEN 2A. We here present, in a Danish couple where the man has MEN 2A, the first European family with children born after PGT-M.

**Objective:** To report the results of PGT-M in relation to multiple endocrine neoplasia type 2A with the aim to increase awareness among physicians treating this and other genetic disorders.

**Methods:** A Danish couple was referred to the PGT Center at Copenhagen University Hospital Rigshospitalet and opted for PGT-M after counseling by a clinical geneticist and a fertility doctor. The embryos were diagnosed using microsatellite polymorphic marker close to *RET*.

**Results:** The couple had two healthy children born in 2017 and 2019 as a result of a total of three ICSI treatments including controlled ovarian stimulation, oocyte retrieval and PGT-M, and a total of six blastocyst transfers.

**Conclusion:** A session with a clinical geneticist covering all reproductive options for patients in early adult life is a relevant part of the clinical management of patients with MEN 2A, and other patients with hereditary cancer predisposition syndromes.

## Introduction

Multiple endocrine neoplasia type 2 (MEN 2) is an autosomal dominant inherited cancer syndrome. MEN 2 is caused by germline mutations of the *RE*arranged during *T*ransfection (*RET)* proto-oncogene (1, 2). The syndrome comprises MEN 2A and MEN 2B. MEN 2A accounts for ~95% of MEN 2 cases and has an estimated point prevalence of 1:42.000–80.000 (3–6). MEN 2A is characterized by the propensity to develop medullary thyroid cancer (MTC), pheochromocytoma, primary hyperparathyroidism, cutaneous lichen amyloidosis and Hirschsprung disease. While virtually all patients develop MTC, the remaining features occur less frequently. Left untreated, MEN 2A has devastating consequences. Accordingly, prophylactic total thyroidectomy is often recommended at an early age. The recommended timing of the surgery is largely dictated by the genotype and by serum calcitonin levels. The intervention follows lifelong monitoring of paraclinical markers (6).

For individuals with MEN 2A, the probability of passing on the pathogenic *RET*-variant to their offspring is 50%. Preimplantation Genetic Testing for Monogenic Disorders (PGT-M) in combination with assisted reproduction technology (ART) is an available reproductive option for couples with MEN 2A. PGT-M is performed to deselect embryos carrying a pathogenic *RET*-variant prior to implantation. In 2015, the American Thyroid Association issued revised guidelines for medullary thyroid carcinoma (6). Here, clinicians were recommended to inform patients with MEN 2 about the availability of PGT-M and refer to a clinical geneticist. Adding to this, patients seem to have a genuine interest in PGT-M in relation to reproduction (7). However, just two cases have been reported so far and none originated from Europe (8, 9). Aiming to increase awareness among physicians treating this and other genetic disorders, especially in Europe, of the possibility for PGT-M in MEN 2A patients, we here report the first European case. Informed written consent has been obtained from the family for publication of the case report.

## Case

In 2000, a Danish family was diagnosed with MEN 2A with Hirschsprung, due to the presence of the *RET*, c.1858T>C, p.C620R germline variant, Hirschsprung's disease and MTC. The family was unrelated to other previously reported Danish families carrying the same variant (10, 11). In this family, a then 11-year old boy tested positive for the *RET* C620R variant. He had a basal calcitonin of 0.42 μg/L (<0.1 μg/L) at the time and underwent prophylactic total thyroidectomy and neck dissection, revealing a T1aN1bM0 MTC. The following four years, the calcitonin level slowly increased to 153 ng/L (<10.5 ng/L) but has now for the last 15 years remained stable despite the fact that no additional treatment has been given.

In 2015, following thorough counseling by a clinical geneticist, the then 26-year old man and his 25-year old partner were referred to the PGT Center at The Copenhagen University Hospital Rigshospitalet, Denmark. His partner was overall healthy. The couple underwent thorough genetic counseling by a clinical geneticist and information about reproductive options, including both PGT-M, spontaneous conception with prenatal diagnosis, sperm donation and adoption was given. The couple decided on PGT-M and was referred to treatment.

In preparation for the genetic analysis of embryo biopsy, a semi-informative microsatellite polymorphic marker was identified (D10S1669). Based on genomic DNA from family members and the female partner, haplotypes were constructed. Linkage analysis was performed using multiplex PCR as previously described (12). Thereby the embryos expecting to carry the C620R variant could be identified and deselected.

Assisted reproduction technology including controlled ovarian stimulation, oocyte retrieval, intracytoplasmic sperm injection (ICSI), *in vitro* culture and embryo biopsy (nowadays blastocyst) is a prerequisite for PGT. In this case, the woman underwent a total of three fresh ICSI treatments that resulted in a total of 25 cleavage stage embryos that could undergo biopsy. After genetic analyses, a total of nine embryos were expected not to carry the pathogenic *RET*-variant.

In November 2017, the first pregnancy was achieved after a total of three ICSI treatments and five blastocyst transfers and resulted in the birth (GA 41+2) of a healthy girl with a birthweight of 3022 g and a length of 50 cm. The couple returned in October 2018 for replacement of one of the two remaining usable blastocysts, which resulted in an ongoing pregnancy and the delivery (GA 41+3) of a healthy girl with a birthweight of 4,290 g and a length of 54 cm. One useable blastocyst remains vitrified. None of the children carry the *RET* C620R variant or suffer from notable illnesses.

## Discussion

When PGT was introduced in 1990, the technology became an alternative approach to traditional prenatal diagnosis in cases where there is a known high risk of a genetically affected fetus due to known familial monogenetic variants (PGT-M) or structural chromosomal rearrangements (PGT-SR) (13–15). Further, PGT can be used to investigate embryos for numerical chromosomal aberrations or aneuploidies (PGT-A) in situations with no known familial genetic disease or as a supplement to PGT-M. Now, PGT is performed on a large scale at specialized facilities worldwide. The technology remains an ethical hot topic, as its use becomes continuously more widespread (16). Importantly, the crucial argument for using PGT lies in the wish to spare future offspring from the burden of a severe disease and NOT in eugenic considerations. Prenatal testing and PGT both focus on deselecting an embryo or fetus carrying a specific disease, rather than treating the disease. A clear advantage of PGT compared to prenatal testing is avoiding the risk of an ethical dilemma in deciding whether to terminate a pregnancy (17). By averting the transmission of a pathogenic *RET*-variant from affected individuals to their offspring, PGT can reduce the disease burden for future generations. Consequently, if applied routinely and consistently as a reproductive option, this technology can limit the population prevalence of MEN 2A, as *de novo* variants are regarded as rare (18). However, so far, the use of PGT in relation to MEN 2A has been limited as there are just two cases reported in the literature. In 2011, Altarescu et al. reported of an Israeli woman with MEN 2A, who by the use of PGT-M, gave birth to genetically unaffected dizygotic twins (8). Chen et al. presented a similar case of PGT-M in a Chinese couple where the male had MEN 2A (9). Unlike some other countries, in Denmark PGT is not reserved for specific diseases for which the procedure has been approved (19, 20). In each individual case the final decision to offer PGT is made by a clinical geneticist and is based on an assessment of the severity of the given disease.

A questionnaire issued by The University of Texas addressed individuals with hereditary cancer syndromes including MEN 2 who had visited a genetic counselor at this institution. Participants were asked about their awareness and acceptance of the PGT (7). Just 24% of the respondents were aware of PGT and individuals of a lower socio-economic status were particularly ill-informed. These results indicate a lack of knowledge among individuals who might stand to benefit from PGT. In addition, 72% felt that PGT should be offered and 43% would consider availing themselves of the technology. An obvious limitation to the study is a low response rate of 38%. However, a comprehensive systematic review with meta-analysis found similar results (21).

Acceptance of PGT may be influenced by multiple sociodemographic and personal variables e.g., age, sex, religious affiliation, health care set-up, income, and medical history (7, 17, 21, 22). Firstly, certain religious beliefs may affect individuals stand on the PGT (7). Religious spokespersons have previously voiced strong opinions against the ethical aspects of PGT and some compare it to abortive practices (23). In many cases, however, PGT is considered an acceptable option, when pregnancy termination, or gamete donation is not compatible with ethical and religious beliefs. Secondly, PGT is a costly procedure which may influence patients' attitudes toward PGT (22). It is likely cost-effective as it may allow for substantial long-term savings being made in diagnosis and treatment (23). The procedure, however, is in some countries not covered by the state or by a standard health insurance. With a success rate of PGT in terms of clinical pregnancy as an outcome measure of ~40% per embryo transfer, multiple cycles are often needed to result in a live birth (15, 24). Lastly, the perception that MEN 2A can be managed by timely surgery with a potentially curative outcome may restrain patients from choosing PGT. Higher levels of acceptance have namely been documented among patients with diseases without available risk-reduction surgery (7). The risk reduction surgery of prophylactic total thyroidectomy is a cornerstone in the management of MEN 2A which is a measure taken to help prevent otherwise almost inevitable cancer disease from occurring (6). However, important points are the facts that this surgical intervention does not protect against other clinical manifestations of MEN 2A other than MTC, it does not remove the risk of persistent or recurring MTC and it is associated with risks of postoperative complications (25–29).

## Conclusion

In this case report, we present the first case of successful PGT-M in MEN 2A in Europe. It may serve to remind clinicians following these patients of the possibility of PGT-M. A session with a clinical geneticist covering all reproductive options for patients in early adult life is a relevant part of the clinical management of patients with MEN 2A, and other patients with hereditary cancer predisposition syndromes.

## Ethics Statement

Written informed consent was obtained from the individual(s) for the publication of any potentially identifiable images or data included in this article.

## Author Contributions

All authors drafted, critically revised, and gave final approval of the manuscript.

## Conflict of Interest

The authors declare that the research was conducted in the absence of any commercial or financial relationships that could be construed as a potential conflict of interest.
